# Contrasting Alleles of *OsNRT1.1b* Fostering Potential in Improving Nitrogen Use Efficiency in Rice

**DOI:** 10.3390/plants13202932

**Published:** 2024-10-19

**Authors:** Jonaliza L. Siangliw, Mathurada Ruangsiri, Cattarin Theerawitaya, Suriyan Cha-um, Wasin Poncheewin, Decha Songtoasesakul, Burin Thunnom, Vinitchan Ruanjaichon, Theerayut Toojinda

**Affiliations:** 1 National Center for Genetic Engineering and Biotechnology (BIOTEC), National Science and Technology Development Agency (NSTDA), Khlong Luang, Pathum Thani 12120, Thailand; mathurada.ruangsiri@gmail.com (M.R.); cattarin.the@biotec.or.th (C.T.); suriyanc@biotec.or.th (S.C.-u.); wasin.pon@biotec.or.th (W.P.); dechakrubb@gmail.com (D.S.); burin.thu@biotec.or.th (B.T.); vinitchan.rua@biotec.or.th (V.R.); 2 Rice Science Center, Kasetsart University, Kamphangsaen, Nakhon Pathom 73140, Thailand; theerayut.to@ku.th

**Keywords:** high-throughput phenotyping, hyperspectral reflectance, nitrogen use efficiency, *OsNRT1.1b*, photosynthetic pigments, rice

## Abstract

Nitrogen use efficiency (NUE) is important for the growth and development of rice and is significant in reducing the costs of rice production. *OsNRT1.1b* is involved in nitrate assimilation, and the alleles at position 21,759,092 on chromosome 10 clearly separate indica (Pathum Thani 1 (PTT1) and Homcholasit (HCS)) and japonica (Azucena and Leum Pua (LP)) rice varieties. Rice morphological and physiological traits were collected at three nitrogen levels (N0 = 0 kg ha^−1^, N7 = 43.75 kg ha^−1^, and N14 = 87.5 kg ha^−1^). Leaf and tiller numbers in PTT1 and HCS at N7 and N14 were two to three times higher than those at N0. At harvest, the biomass yield in PTT1 was the highest, while the total grain number in HCS was the maximum. The leaf widths and total chlorophyll contents (SPAD units) of Azucena and LP increased with nitrogen application as well as photosynthetic pigment parameters; for example, plant senescence reflectance indices (PSRIs), structure-insensitive pigment indices (SIPIs), and modified chlorophyll absorption ratio indices (MCARIs) were highly related in the japonica varieties. PTT1 and HCS, both carrying the A allele at *OsNRT1.1b*, had better NUE than Azucena and LP with the G allele. HCS, overall, had better NUE than PTT1. The translation to grain yield of assimilates was remarkable in PTT1 and HCS compared with Azucena and LP. In addition, HCS converted biomass for a 75% higher yield than PTT1. The ability of HCS to produce high yields was achieved even at N7 nitrogen fertilization, manifesting efficient use of nitrogen.

## 1. Introduction

Nitrogen is a crucial element for the growth and development of rice plants. Nitrogen is a fundamental component of amino acids, the building blocks of proteins. Proteins are essential for the structural and functional components of rice plants, including enzymes that drive metabolic processes necessary for growth [1]. Adequate nitrogen levels ensure the production of sufficient chlorophyll, allowing rice plants to effectively convert sunlight into energy and, consequently, promoting healthy growth and high yields [2]. Nitrogen influences the development of leaves and stems, which are crucial for capturing sunlight and supporting the plant structure. An optimal nitrogen supply results in taller plants with more tillers (shoots) and leaves, enhancing the overall biomass [3]. The application of nitrogen fertilizers is directly linked to the yield and quality of rice grains. Nitrogen influences the number of grains per panicle (the flowering part of the plant) and the grain size, which are critical determinants of the harvest quantity [4,5].

In Thailand, the recommended nitrogen application depends on soil type and rice photo-period sensitivity. A photo-period-insensitive rice variety planted in clay soil needs two rounds of fertilizer application. The first application at 15–20 days after transplanting uses 16-20-0 formula at 187.5 kg ha^−1^, and the second application at 25 to 30 days before flowering or at 50 to 60 days after seeding uses 46-0-0 at 125 kg ha^−1^ [6]. All in all, the total amount of nitrogen applied is 87.5 kg ha^−1^. In Thailand, particularly in irrigated areas, rice can be planted in two to three crop cycles per annum; thus, nitrogen application may be increased from the recommended 87.5 kg ha^−1^ to intensify production, which may lead to deleterious effects on the environment. Nitrogen use efficiency (NUE) is critically important in rice cultivation. Efficient use of nitrogen fertilizers can significantly reduce the cost of rice production. Fertilizers represent a substantial portion of the total production cost, so improving NUE helps farmers achieve better yields without increasing fertilizer inputs, leading to higher profitability [7]. Excessive use of nitrogen fertilizers can lead to environmental problems such as water pollution and greenhouse gas emissions [8,9]. Improving NUE reduces these emissions and maintains soil health by preventing the buildup of excess nitrogen in the soil—a key component of sustainable agricultural practices. Higher NUE means that rice plants can utilize nitrogen more effectively, leading to better growth, higher yields, and improved grain quality.

*OsNRT1.1b* (also known as *OsNPF6.5*) encodes a nitrate transporter and is one of the genes involved in nitrogen assimilation. The transporter is a protein responsible for the uptake and transport of nitrate (NO_3_^−^) from the soil into the plant roots and its translocation within the plant [10]. It has dual affinity and thus can efficiently take up nitrate at both low and high concentrations. By efficiently transporting nitrate, *OsNRT1.1*b helps the plant make better use of available nitrogen in the soil. This enhances nitrogen use efficiency (NUE), reducing the need for excessive nitrogen fertilizer application. Understanding the role of *OsNRT1.1b* allows researchers to target this gene for genetic improvement programs [10]. By breeding or genetically engineering rice varieties with enhanced *OsNRT1.1b* function, it is possible to develop crops with better nitrogen uptake efficiency. *Indica* and *japonica* rice have different alleles of the *OsNRT1.1b* gene. Specific nucleotide differences in the *OsNRT1.1b* gene sequence between *indica* and *japonica* rice contribute to variations in nitrate uptake and transport efficiency [11,12].

A high-throughput phenotyping system is a platform designed for non-destructive measurement, big data collection, and precision plant phenotypic analysis of whole life cycles. This system combines modern imaging technologies, automated handling, and powerful data analysis tools to enable detailed and large-scale research on plant features under a variety of environmental circumstances [13]. It has several imaging modules, including RGB (red–green–blue), hyperspectral, thermal, chlorophyll fluorescence, and 3D (three-dimensional) imageries. The approach accelerates the identification of desirable features and the selection of superior genotypes, hence boosting crop development efforts. Furthermore, it helps researchers understand how plants react to diverse biotic and abiotic stimuli, which aids in the development of stress-resistant cultivars. It also integrates phenotypic data and genetic information, improving our understanding of gene activity and control [14,15]. Finally, precise phenotypic information aids precision farming operations by optimizing inputs such as water and fertilizers based on plant health and performance, making this approach useful in understanding precision agriculture [16]. Recently, a close correlation between nitrogen level and chlorophyll stabilization in rice canopies has been well established using hyperspectral reflectance [17,18,19]. Based on basic knowledge, the chlorophyll degradation in relation to hyperspectral-based vegetation indices under different nitrogen levels is an interesting issue for optimum nitrogen supplementation in rice fields.

In this study, we used rice varieties varying in single-nucleotide polymorphisms (SNPs) in *OsNRT1.1b* at position 21,759,092 on chromosome 10. *Indica* varieties carry the A allele, while *japonica* varieties have the G allele. The morphological and physiological responses of the rice varieties obtained by plant phenotyping systems under different nitrogen levels were collected and used to identify the traits highly associated with *OsNRT1.1b* and response to nitrogen use efficiency as expressed by increase in yield compared to zero nitrogen application.

## 2. Results

### 2.1. Response of Rice at Different Nitrogen Levels

**Figure 1:**
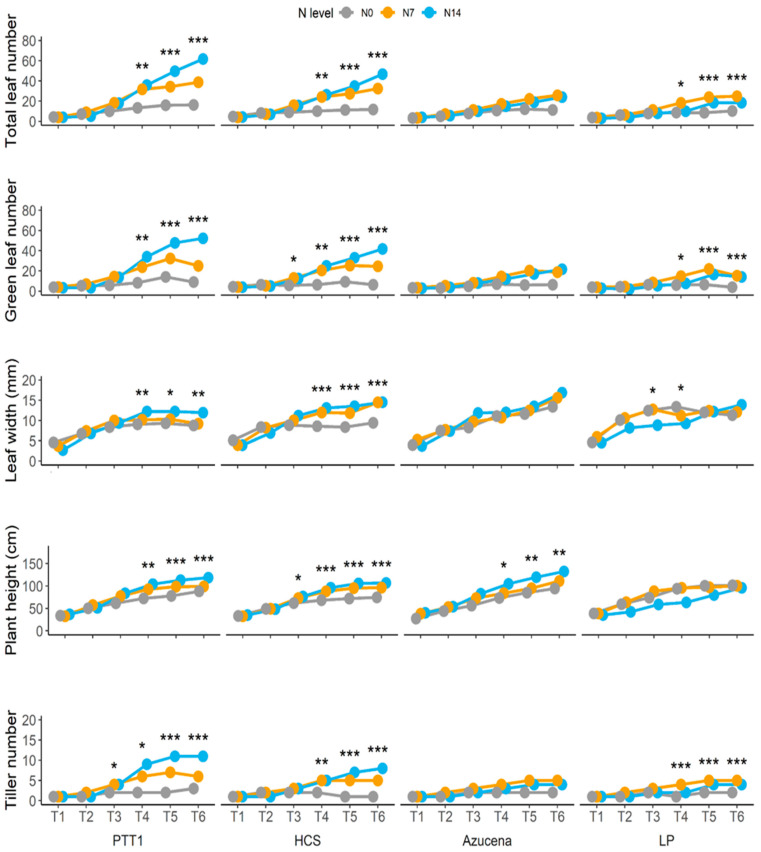
Agronomic response of different rice varieties (PTT1, HCS, Azucena, and LP) at different times (30 DAS (seedling) (T1), 45 DAS (T2), 58 DAS (T3), 72 DAS (T4), 86 DAS (T5), and 100 DAS (grain filling) (T6)) and nitrogen levels (N0 (0 kg/ha), N7 (43.8 kg/ha), and N14 (87.5 kg/ha)). *, **, and *** represent significant differences at 0.05, 0.01, and 0.001 levels, respectively.

In determining the response of rice at different nitrogen levels, several traits were measured and collected, including morphological and physiological traits. Each rice variety responded positively to the nitrogen application at both 87.5 kg ha^−1^ (N14) and 43.75 kg ha^−1^ (N7) levels. Total leaf and green leaf numbers increased as the nitrogen applied increased. At 72 days after seeding (or T4), the increase in leaf number was highly distinct from the control (N0), with PTT1 and HCS showing 171% and 158% increases at N14, respectively, and both varieties showing a 136% increase at N7 (Figure 1). The green leaf numbers out of the total leaf numbers were found to be significantly increased at T4 to T6 in PTT1 and HCS, with four to six times higher leaf numbers at N14 and two to four times higher numbers at N7 (Figure 1). Increase in leaf number was also observed in Azucena and LP but was significantly lesser—by two or three times—than in PTT1 and HCS. The same response was found for leaf width, with both PTT1 and HCS at N14 showing a significant increase in leaf width—between 35% (at T4) and 36% (T6) in PTT1 and between 52% (T4) and 54% (at T6) in HCS—compared to the N0 treatment (Figure 1). Although the highest leaf width recorded was from Azucena, the increase in leaf width at N14 and N7 was not significantly different from the leaf width at N0, and the same was true for LP. Plant height in PTT1 and HCS was also increased because of the nitrogen application. At N14, an approximately 40% increase in plant height was found in PTT1 and HCS, and about a 12% to 30% increase was found at N7 (Figure 1). An increase in plant height at N14 was also found in Azucena, with around a 40% difference in the N0 condition and a 12 to 20% difference in the N7 condition. Increases in tiller numbers were obviously found in PTT1 and HCS under both the N14 and N7 conditions (Figure 1). LP also showed an increase in tiller number two to three times higher than in the N0 condition (Figure 1).

**Figure 2:**
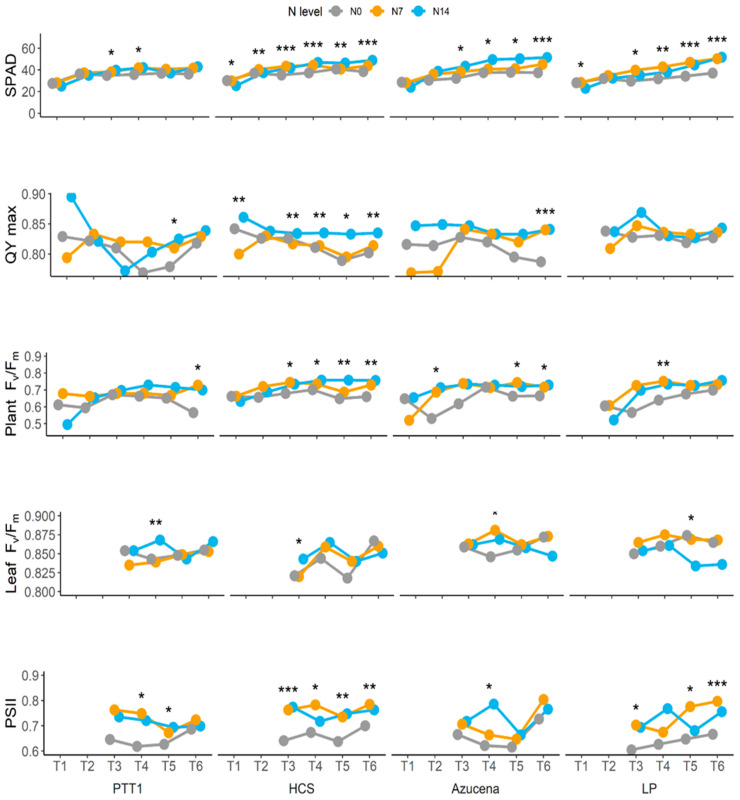
Relative chlorophyll content (SPAD) and chlorophyll fluorescence parameter response of four rice varieties (PTT1, HCS, Azucena, and LP) at different growth stages (30 DAS (seedling) (T1), 45 DAS (T2), 58 DAS (T3), 72 DAS (T4), 86 DAS (T5), and 100 DAS (grain filling) (T6)) and nitrogen levels (N0 (0 kg/ha), N7 (43.8 kg/ha), and N14 (87.5 kg/ha)). *, **, and *** represent significant differences at 0.05, 0.01, and 0.001 levels, respectively.

Relative chlorophyll content measured using SPAD increases as rice ages. At N14, Azucena showed huge differences to N0, reaching 35 to 55%. Leum Pua also showed an improved chlorophyll content, followed by Homcholasit (Figure 2). QY max, used in measuring the efficiency of the photosynthetic apparatus in converting light energy to chemical energy, was measured using the phenomics system. PTT1 and HCS were efficient among the varieties tested, their QY max values being 3 to 6% higher compared with the N0 level. The same was true for F_v_/F_m_, also measured by the phenomics system. N14 and N7 had higher F_v_/F_m_ values than the N0 condition, and better F_v_/F_m_ results for HCS were recorded at T4 (72 DAS), T5 (86 DAS), and T6 (100 DAS), with an 8% to 17% increase compared with N0 (Figure 2). The effective quantum yield of PSII also showed an increase at N14 and N7, particularly in HCS, in which it was higher than at N0 (Figure 2). The rate of photosynthesis was higher in N14 for HCS and LP, particularly at T4 (72 DAS), while PTT1 had the lowest rate. HCS maintained a high photosynthesis rate until T6 (100 DAS), and the lowest rate was recorded for Azucena (Figure S1). HSC had the highest stomatal conductance and transpiration at both 87.5 kg ha^−1^ (N14) and 43.75 kg ha^−1^ (N7), signifying the importance of nitrogen in the process of photosynthesis in rice.

Several parameters determined by the hyperspectral camera were measured at T5 and T6. The plant senescence reflectance index (PSRI) measures senescence and stress in a plant. PSRI at T5 for 87.5 kg ha^−1^ (N14) and 43.75 kg ha^−1^ (N7) was high in HCS compared to the N0 level (Supplementary Figure S2), but PSRI decreased at T6. On the other hand, Azucena and LP showed an increase in PSRI from T5 to T6, probably due to higher chlorophyll contents. The photochemical reflectance index (PRI) measures photosynthesis efficiency and stress response. PRI in HCS was maintained in T5 and T6, while the PRI in PTT1 was the lowest among the varieties tested (Figure S2). In the case of Azucena and LP, a decrease in PRI in N7 indicated a deficiency in nitrogen (Figure S2). The simple ration pigment index (SIPI) clearly showed pigment deficiency at T6 in both PTT1 and HCS, while it was not limited in Azucena and LP (Figure S2). The modified chlorophyll absorption in reflectance index (MCARI) showed a decrease from T5 to T6 in PTT1 and HCS compared with Azucena and LP. One interesting response found in PTT1 and HCS was a higher MCARI at N0 compared to N14 and N7.

**Figure 3:**
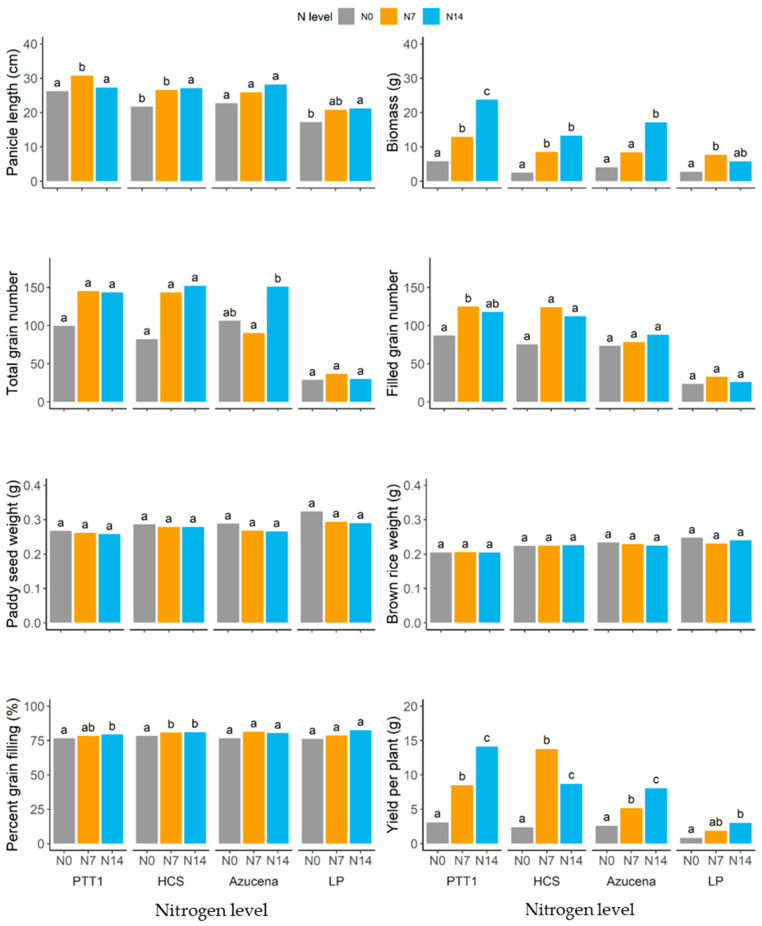
Yield and yield components of different rice varieties (PTT1, HCS, Azucena, and LP) at different nitrogen levels (N0 (0 kg/ha), N7 (43.8 kg/ha), and N14 (87.5 kg/ha)) at harvest. Small letters (abc) represent significant differences of the traits at different nitrogen levels for each variety.

At harvest, several traits were obtained to assess the productivity of the rice varieties under the different nitrogen levels. Panicle length was significantly increased in PTT1 and HCS at 87.5 kg ha^−1^ (N14) and 43.75 kg ha^−1^ (N7), while nitrogen did not promote panicle length elongation in Azucena and LP (Figure 3). Biomass yield at N14 and N7 was highest in PTT1, while LP had the lowest yield. It is interesting to note that nitrogen also promoted biomass growth in Azucena, which could have been due to an increase in plant height. It was interesting that nitrogen promoted an increase in spikelet number (TGN) in both PTT1 and HCS at N14 and N7 and in Azucena (only at N14), while LP did not show an increase in spikelet number. Considering only the number of filled grains, both PTT1 and HCS had more filled grains than Azucena and LP (Figure 3). The weight of the paddy seed and brown rice were not different at all nitrogen levels in all rice varieties, but it is interesting that the grain filling, which is the percentage brown rice weight per paddy seed weight, was high in Azucena and LP, despite low spikelet numbers, particularly in LP. But although the percentage grain filling was high in LP, the yield per plant was the lowest due to the low number of spikelets per panicle, and it was evident that the high yield per plant in PTT1 and HCS was supported by the number of grains (and filled grains) per panicle. PTT1 had a higher yield per plant at 87.5 kg ha^−1^ (N14), while HCS had a better yield at 43.75 kg ha^−1^ (N7).

### 2.2. Response of Allele Groups of Rice Varieties at T6 and at Harvest

**Figure 4:**
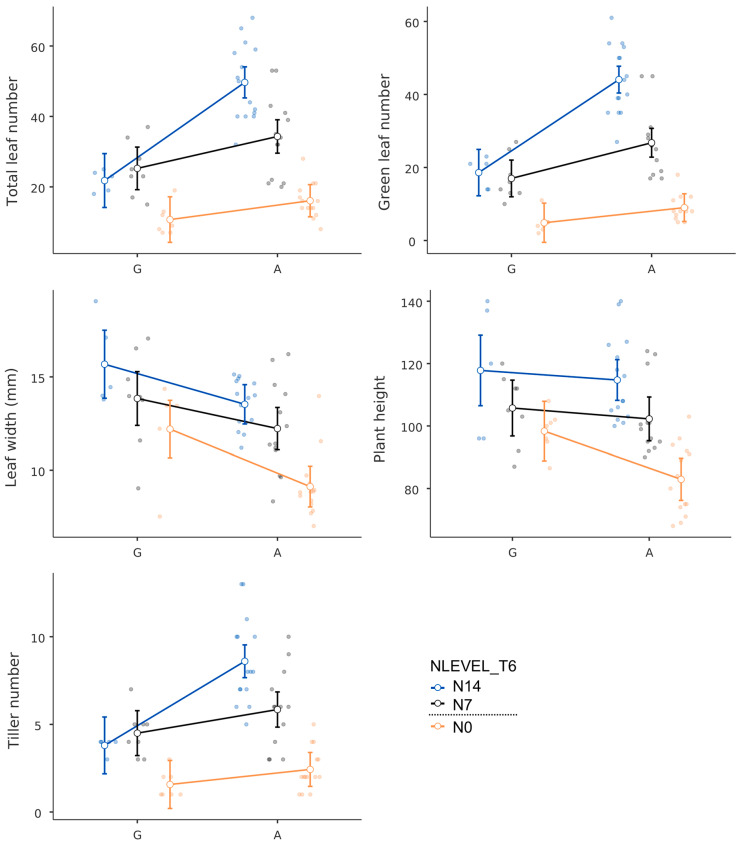
Agronomic response of rice varieties carrying G and A alleles at *OsNRT1.1b* at different nitrogen levels (N0 (0 kg/ha), N7 (43.8 kg/ha), and N14 (87.5 kg/ha)).

The rice varieties used were grouped as those carrying the A allele of *OsNRT1.1b*, namely, PTT1 and HCS, and those carrying the G allele, namely, Azucena and LP. Those carrying the A allele had higher total leaf and green leaf numbers and had higher tiller numbers. On the other hand, Azucena and LP had wider leaves and taller plant heights (Figure 4).

**Figure 5:**
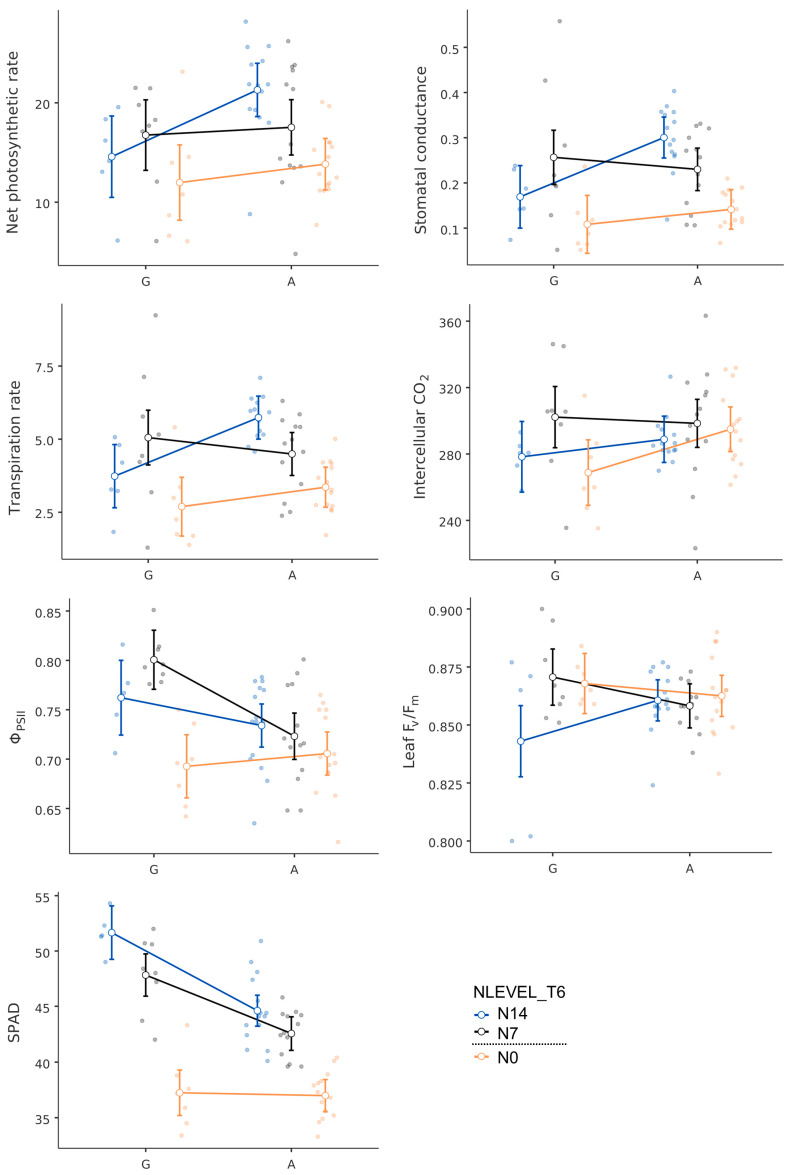
Physiological responses measured by LI-COR 6400 XT, SPAD, and leaf chlorophyl fluorescence of rice varieties carrying the G and A alleles at *OsNRT1.1b* at different nitrogen levels (N0 (0 kg/ha), N7 (43.8 kg/ha), and N14 (87.5 kg/ha)).

**Figure 6:**
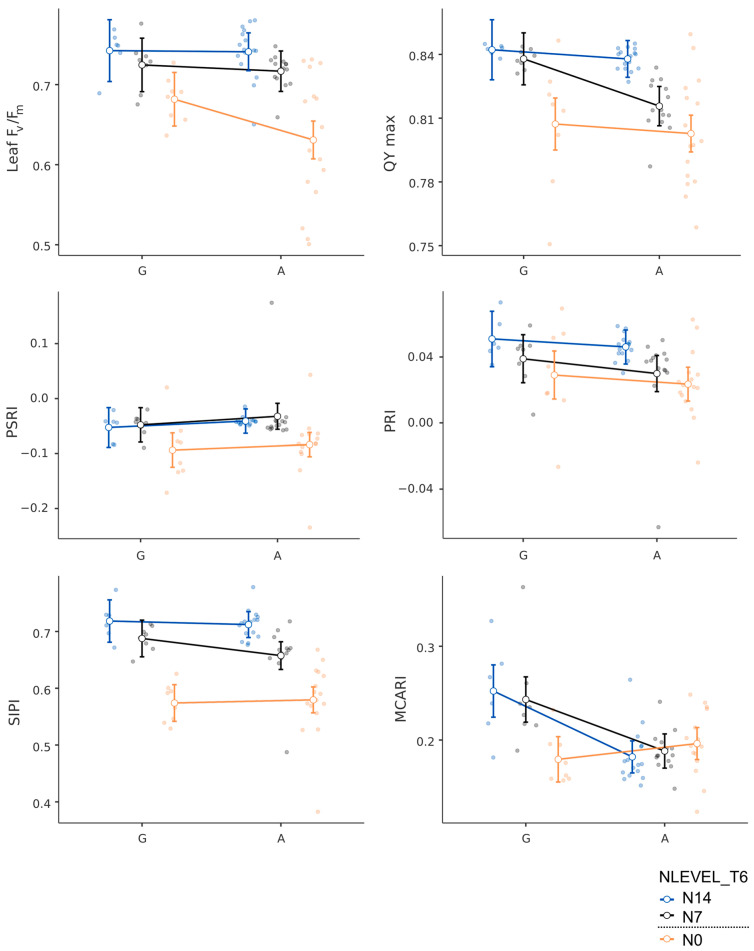
Hyperspectral reflectance parameters of rice varieties carrying the G and A alleles at *OsNRT1.1b* at different nitrogen levels (N0 (0 kg/ha), N7 (43.8 kg/ha), and N14 (87.5 kg/ha)).

The rice varieties with the G allele had a higher chlorophyll content based on SPAD than those carrying the A allele. Due to this, the plant and leaf F_v_/F_m_ values detected, as well as the QY max values, showed that the G allele-carrying rice varieties had higher values, except for the effective quantum yield of PSII, which was higher in the A allele-carrying rice varieties (Figure 5). Rates of photosynthesis, stomatal conductance, and transpiration were higher in Azucena and LP, the G allele-carrying rice varieties, but at T6, the A allele-carrying rice varieties had higher measurements (Figure 6). In support of the findings, PSRIs, PRIs, SIPIs, and MCARIs were higher in the G allele group (Figure 6).

**Figure 7:**
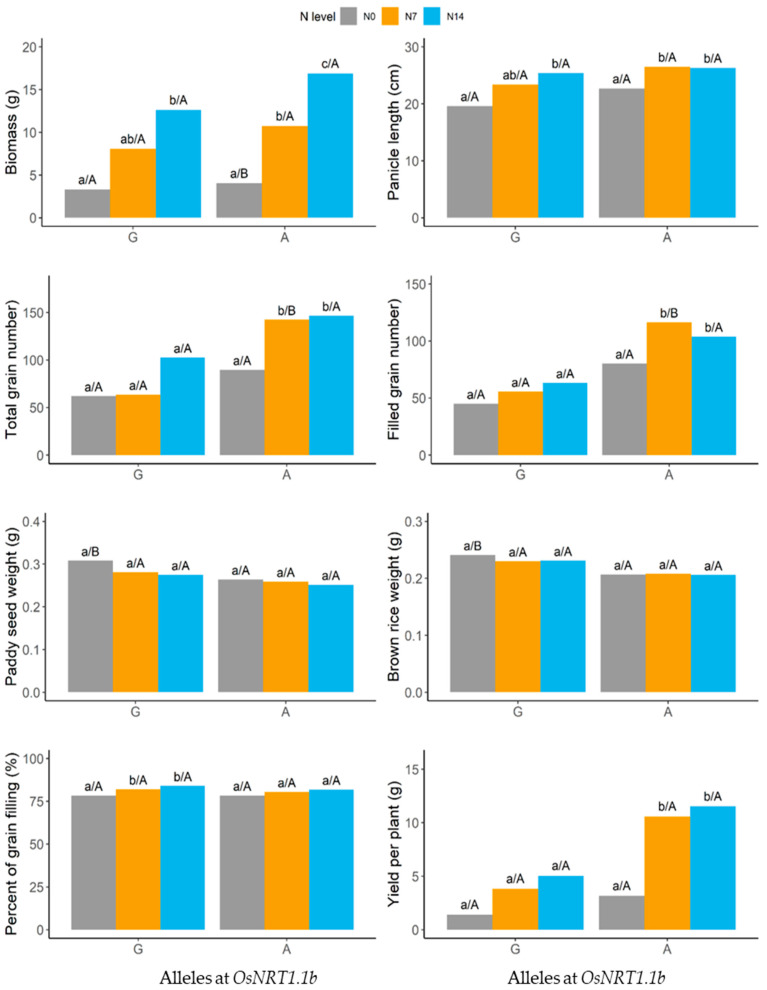
Yields and yield components of rice varieties carrying the G and A alleles at *OsNRT1.1b* at different nitrogen levels (N0 (0 kg/ha), N7 (43.8 kg/ha), and N14 (87.5 kg/ha)). Small letters represent significant differences of the trait at different nitrogen levels with the same allele, while capital letters represent significant differences of the trait at different nitrogen levels between two alleles.

At harvest, PTT1 and HCS—the rice varieties with the A allele—had longer panicles, higher biomass yields, higher total grain and filled grain numbers, and higher yields per plant. The paddy seed and brown rice weights were higher in the G allele group, making the results interesting (Figure 7).

### 2.3. Correlation of Productivity Traits and NUE with Morphological and Physiological Parameters Determining Growth Under 43.75 kg ha^−1^ (N7) and 87.5 kg ha^−1^ (N14) Nitrogen Applications

**Figure 8:**
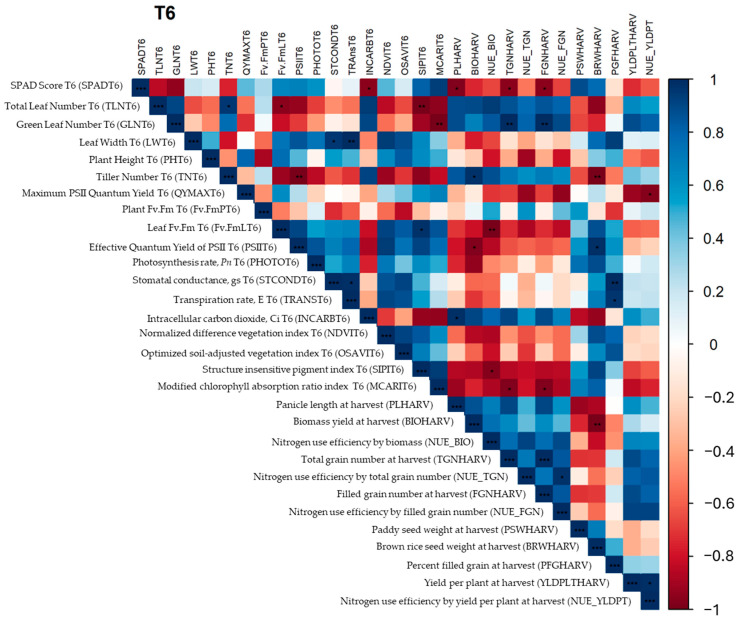
Correlation matrix of morphological and physiological traits at N7 (43.8 kg/ha) nitrogen level among the lines tested. Positive and negative correlations are shown with blue and red squares, respectively. Color shading is proportional to the correlation coefficients, with their values corresponding to the color intensity bar. Significance levels are indicated with asterisks (*p* < 0.05 *; *p* < 0.01 **; *p* < 0.001 ***).

The correlation of traits under N7 collected at T6 and at harvest was determined. Yield and yield components showed correlation with agronomic and physiological traits. Biomass yield was positively correlated with tiller number and negatively correlated with brown rice weight and the effective quantum yield of PSII. It is interesting that an increase in biomass may result in resource allocation trade-offs. F_v_/F_m_ is not the sole indicator of the efficiency of photosynthesis; thus, the negative correlation of F_v_/F_m_ and NUE based on biomass yield may be prioritized and reduce investment to maintain PSII as measured by F_v_/F_m_. The negative correlation between SIPI and NUE based on biomass yield reflects reduced PSII efficiency and the prioritization of biomass production over maintaining maximum photosynthetic efficiency. Yield components like panicle length are positively correlated with intercellular CO_2_ but negatively correlated with relative chlorophyll measured by SPAD. The available CO_2_ may enhance photosynthesis and provide more energy and build blocks for growth, including the development of long panicles. On the other hand, chlorophyll production, which is very dependent on nitrogen, and limited available nitrogen may cause preferential development of panicles rather than maintenance of the chlorophyll content in leaves. Total grain number was found to be dependent on green leaf number, while low SPAD values and MCARIs may indicate plant health, particularly chlorophyll concentrations, and thus the reallocation of nitrogen from the leaves to the grains. This correlation also applies to the filled grain number. A negative correlation between brown rice weight and tiller number was found, indicating the preferential allocation of resources. On the other hand, the positive correlation of brown rice weight and effective quantum yield of PSII under 43.75 kg ha^−1^ (N7) nitrogen application indicates efficient production of photosynthates for grain filling and development. Efficient gas exchange supports photosynthesis and nutrient transport, contributing to higher grain filling. PSRI is used to detect plant senescence and thus the breakdown of chlorophyll; thus, a higher PSRI may indicate shorter periods of photosynthesis and nutrient supply to the grain. A negative correlation was found between PSRI and percent grain filling (Figure 8).

**Figure 9:**
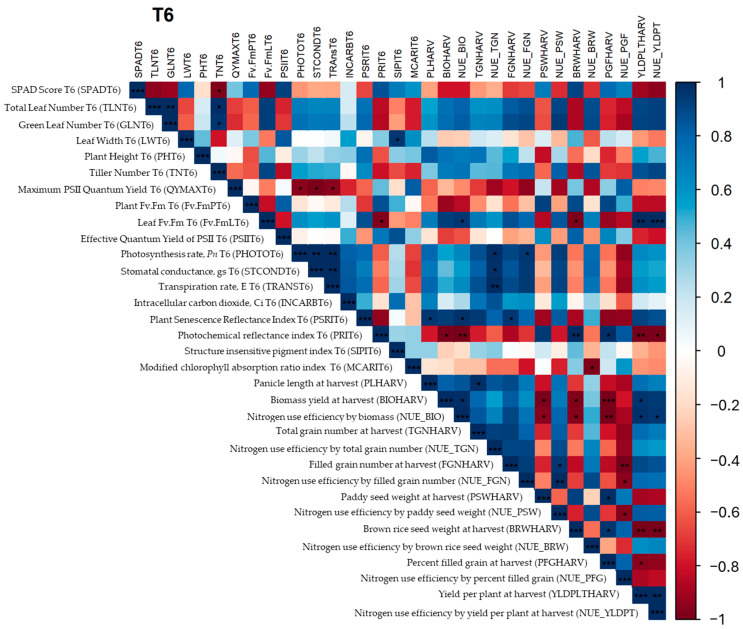
Correlation matrix of morphological and physiological traits at the N14 (87.5 kg/ha) nitrogen level among the lines tested. Positive and negative correlations are shown with blue and red squares, respectively. Color shading is proportional to the correlation coefficients, with their values corresponding to the color intensity bar. Significance levels are indicated with asterisks (*p* < 0.05 *; *p* < 0.01 **; *p* < 0.001 ***).

Under the 87.5 kg ha^−1^ (N14) nitrogen application, the correlations of morphological and physiological traits with productivity and NUE were determined. High F_v_/F_m_ indicates lower levels of stress and better overall plant health, therefore supporting higher photosynthetic rates, translating into higher yields per plant. On the other hand, the negative correlation between yield per plant and PRI may indicate an effective management of light energy for photosynthesis which supports growth and yield. This relationship also applies with NUE based on yield per plant. High-biomass plants may allocate more carbohydrates to support vegetative growth rather than grain filling. This can result in a lower percentage of grain filling, as the grains receive fewer resources, resulting in lower paddy seed and brown rice weights. Higher biomass might be associated with increased metabolic activity and potential stress, leading to higher PRI values, which indicate greater non-photochemical quenching. Plants with high biomass are often more efficient in nutrient uptake and utilization, supporting both vegetative and reproductive growth, resulting in higher yields per plant. Overall, NUE based on biomass had a similar relationship with traits associated with biomass yield, and, in addition, high F_v_/F_m_ supports higher photosynthesis and increased biomass yield. Adequate nitrogen may delay senescence (high PSRI) and therefore support growth, leading to plant development, including longer panicles. NUE based on total number of grains or spikelets was found to be positively correlated with photosynthesis rate, stomatal conductance, and transpiration. NUE based on total grain number measures the efficiency with which a plant converts available nitrogen into grain production. Adequate nitrogen supply enhances the photosynthetic capacity of plants. High photosynthesis rates mean that more carbohydrates are produced, supporting greater grain production. Thus, plants with higher NUE based on total grain number also have higher photosynthesis rates because they efficiently convert nitrogen into photosynthetic machinery and activity. High-NUE plants maintain high stomatal conductance to facilitate adequate CO_2_ uptake for photosynthesis. High transpiration rates are often associated with high stomatal conductance and photosynthetic rates. Plants with high NUE ensure that transpiration supports nutrient transport and cooling, optimizing conditions for high photosynthetic activity and grain production. Under adequate nitrogen supply, plants might still face trade-offs between maintaining high photosynthetic efficiency (F_v_/F_m_) and reallocating resources towards grain filling or brown rice weight. During the grain filling stage, plants may prioritize reproductive success by redirecting resources from photosynthetic tissues to grains, leading to lower F_v_/F_m_ values but higher brown rice weights. When more resources are directed to developing grains, photosynthetic efficiency might decrease slightly. Higher PRI values indicate senescence and stress, during which resources are mobilized to support grain filling, resulting in increased brown rice weight. Plants achieving higher yields per plant might spread resources across more grains, potentially reducing the percent grain filling per individual grain. Higher PRI values suggest that plants are undergoing senescence or stress. During senescence, nutrients and carbohydrates are redirected from vegetative tissues to developing grains, promoting higher percent grain filling (Figure 9).

### 2.4. Principal Component Analysis (PCA)

**Figure 10:**
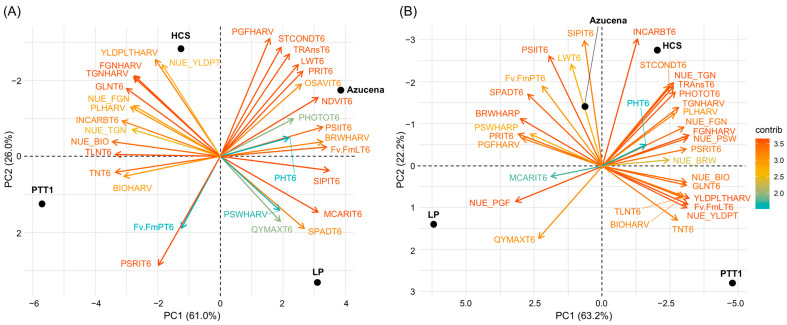
Trait loading scores of the morphological and physiological traits for each principal component under two nitrogen levels: (**A**) N7 (43.8 kg/ha) and (**B**) N14 (87.5 kg/ha).

Three PCs were identified in the N7 condition. The first PC, which had an eigen value of 15.9, explained 61% of the variations in the varieties tested under the 43.7 kg ha^−1^ (N7) condition. PC1 included productivity traits, such as yield per plant (YLDPLT), biomass yield (BIO), total grain and filled grain numbers (TGN and FGN), tiller number (TN), and total and green leaf numbers (TLN and GLN). Intercellular CO_2_ concentration was also positively correlated with productivity traits. Pigment traits, such as relative chlorophyll content by SPAD, the structure-insensitive pigment index (SIPI), and the modified chlorophyll absorption in reflectance index (MCARI), together with the grain filling traits, namely, paddy seed weight (PSW) and brown rice weight (BRW), were also included in PC1 but in the opposite direction to the traits mentioned above. PC2, which explained 26% of the variance, included photosynthesis (PHOTO) and gas exchange traits (STCOND and TRANS), as well as the effective quantum yield of PSII (Φ_PSII_), leaf width (LW), and the photochemical reflectance index (PRI), among the traits that are in the same direction. The plant senescence reflectance index (PSRI) was in the opposite direction to the other traits in PC2. In PC3, plant height (PH) and QY max, together with plant F_v_/F_m_, which was in the opposite direction to PH and QY max, explained 13% of the variance. PTT1 was found to be associated with biomass yield (BIO), total leaf number (TLN), and tiller number (TN), but brown rice weight (BRW), percent grain filling (PGF), photosynthesis rate (PHOTO), and gas exchange traits were found to be not associated with PTT1. HCS, on the other hand, showed high yield per plant (YLDPLT) and total and filled grain numbers (TGN and FGN), but it was not associated with paddy seed weight (PSW) or chlorophyll-related traits. Azucena had a high association with leaf width (LW) and grain filling traits (PGF and BRW), but it was not associated with biomass production. Leum Pua was associated with chlorophyll-related traits (SPAD, SIPI, and MCARI) and paddy seed weight but not with panicle features, including grain number (Figure 10A).

At N14, three principal components were identified. The first PC included productivity traits, such as the overall yield per plant (YLDPLT), total grain number (TGN), and panicle length (PL). Plant height (PH) was also included, together with biomass yield (BIO) and physiological traits such as leaf F_v_/F_m_ (F_v_/F_m_L) and the plant senescence reflectance index (PSRI). Those traits, however, were in opposite directions to paddy seed weight (PDW), brown rice weight (BRW), percent grain filling (PGF), and the photochemical reflectance index (PRI). PC1 had an eigen value of 16.4 and explained 63.2% of the total variations found in the varieties tested. PC2, on the other hand, explained 22.2% of the variation and included photosynthesis and gas exchange traits, such as photosynthesis rate (PHOTO), stomatal conductance (STCOND), transpiration rate (TRANS), and intercellular CO_2_ (INCARB). Also, filled grain number (FGN) was included in PC2 and had the same direction as the traits above. QY max (QYMAX) and the modified chlorophyll absorption in reflectance index (MCARI) were in opposite directions to the traits in PC2. The last principal component, PC3, included leaf width (LW), effective quantum yield of PSII (ΦPSII), relative chlorophyll content measured by SPAD (SPAD), and the structure-insensitive pigment index (SIPI), wherein those traits were related with chlorophyll. These traits were in the opposite direction to tiller number (TN) and total leaf and green leaf numbers (TLN and GLN). PC3 explained 14.6% of the total variation identified. PTT1 was highly associated with traits such as tiller number, biomass yield, total leaf number, yield per plant, and leaf F_v_/F_m_. In contrast, traits related to grain filling and paddy and brown rice weights were not associated with PTT1. HCS, on the other hand, was associated with total grain and filled grain numbers. It was highly associated with photosynthesis and gas exchange parameters, including intercellular CO_2_. HCS was negatively associated with QY max. Traits such as leaf width and pigmentation (SIPI and SPAD) and grain filling traits (PGF, PSW, and BRW) were among the traits associated with Azucena and biomass production traits; tiller and leaf numbers do not contribute to the productivity of Azucena. Leum Pua, although highly associated with QY max, may not be able to convert products of photosynthesis for growth and yield production, but somehow it is associated with grain filling traits, particularly paddy seed weight (Figure 10B).

### 2.5. Nitrogen Use Efficiency (NUE)

**Figure 11:**
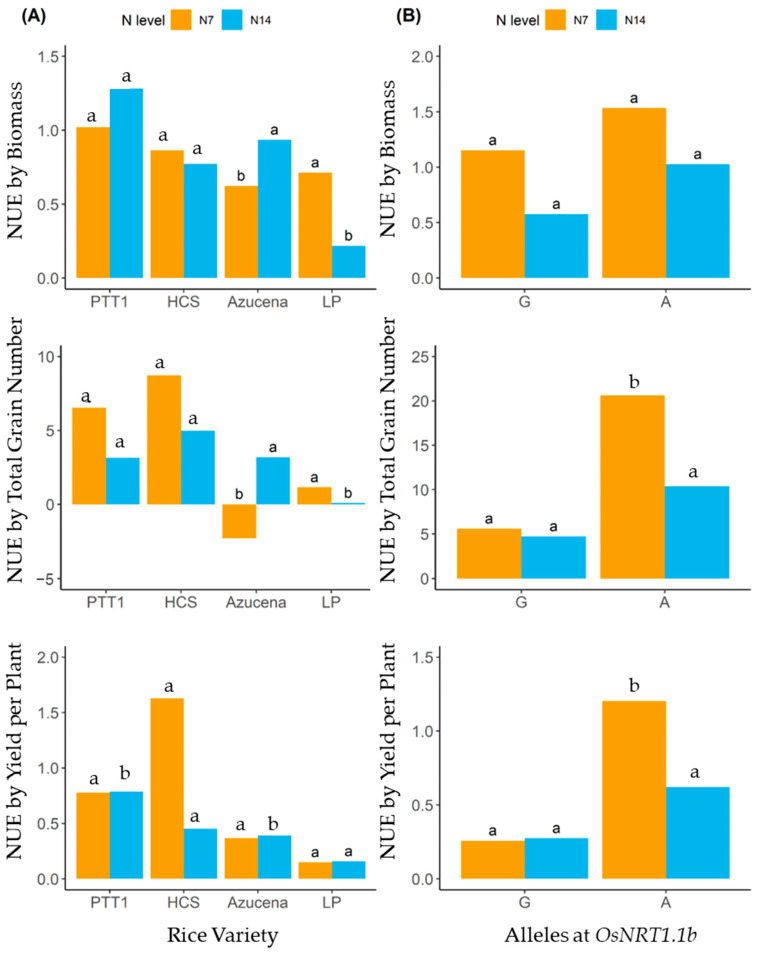
Nitrogen use efficiency based on biomass (BIO), total grain number (TGN), and yield per plant (YLDPLT) of (**A**) varieties carrying the A allele (PTT1 and HCS) and the G allele (Azucena and LP) of *OsNRT1.1b* and (**B**) allele groups (A and G). Small letters represent significant differences in NUE at N7 and N14 in each rice variety (**A**,**B**) differences in NUE between alleles at the same nitrogen level.

Nitrogen use efficiency was determined using biomass yield, total grain number, and yield per plant. PTT1 had the highest NUE based on biomass (NUE_BIO) yield at both 43.75 kg ha^−1^ (N7) and 87.5 kg ha^−1^ (N14) levels, while HCS and LP had higher NUE_BIO at N7 and Azucena had a higher NUE_BIO at N14 (Figure 11A). LP at N14 did not result in an increase in biomass because, as seen in the response to different nitrogen levels, LP had the very lowest increase in leaf and tiller numbers. NUE based on total grain number (NUE_TGN) was higher in HCS compared with PTT1. While both PTT1 and HCS have the A allele of *OsNRT1.1b*, translating the biomass to grain yield was more effective in HCS. It is worth noting that both PTT1 and HCS showed higher NUE_TGN at N7 than at N14. Both Azucena and LP showed inefficiency in converting photosynthates into grain yield (Figure 11A). HCS had the highest NUE based on yield per plant (NUE_YLDPLT) at N7, while PTT1 did not show any difference in NUE_YLDPLT at both N7 and N14. Nevertheless, PTT1 and HCS had higher NUE_YLDPLT than Azucena and LP (Figure 11A).

Nitrogen use efficiency based on biomass yield, total grain number, and yield per plant was highest at 43.75 kg ha^−1^ (N7) in varieties carrying the A allele (Figure 11B). This is an indication that fertilization at N7 is enough to result in even better yields than at 87.5 kg ha^−1^ or N14 rate. However, the recommended rate, 87.5 kg ha^−1^ (N14), resulted in high NUE in the A allele-carrying varieties, and it was shown clearly that the G allele is associated with low yield and inefficiency in utilizing resources for growth and grain yield production.

## 3. Discussion

### 3.1. Rice Response at Different Nitrogen Levels and Effects of Alleles of OsNRT1.1b

Nitrogen is important for rice growth because it is an essential component of biomolecules, such as nucleotides, amino acids, and chlorophyll, and is essential for the development of rice [20]. The leaf number plays a significant role in crop leaf area development and determines photosynthetic activities and crop growth at the end [21]. In a study of two rice types, hybrid *indica* and *japonica*, rates of nitrogen applications significantly influenced the leaf number of hybrid *indica*, and the *japonica* rice was not responsive to the nitrogen applications [22,23]. In this study, the application of nitrogen in amounts of 87.5 kg ha^−1^ (N14) and 43.75 kg ha^−1^ (N7) promoted an increase in leaf number, particularly in *indica* rice varieties, namely, PTT1 and HCS, and the highest increase in leaf number in those varieties was identified at T3 or nearly 60 DAS, with a 125 to 240% increase at N14 and a 100 to 125% increase at N7. Nitrogen is also known to promote cell division and enlargement; thus, in addition to the increase in leaf number, leaf width is also influenced by nitrogen. Prior research has found that leaf width not only affects the morphogenesis of leaves but serves a primary role in the regulation of grain and panicle traits [24]. Zhu et al. [25] identified a rice mutant, *leaf width 5* (*lw5*), that displayed small grains and wide leaves typical of small sink and large source characteristics. Azucena and LP are among the varieties that have wide leaves and small grains. It can be speculated that Azucena and LP are *lw5*; thus, it may be inherent to these varieties to have wide leaves and small grains. Plant height and tiller number are growth parameters in rice that also respond to nitrogen applications. The highest increase in plant height between 58% and 63% in PTT1 and HCS was observed at T3 at N14, while Azucena and LP increased by 39 to 54% at the same nitrogen level. At 43.75 kg ha^−1^ (N7), an increase was more evident at T2 (45 DAS), with an increase in plant height of 52 to 77% in PTT1 and HCS and an increase of 38 to 64% in Azucena and LP. The same observations were made for tiller number, the highest increase in tiller number being observed at T3 at N14 and T2 at N7. Kumar et al. [26] identified a 50 up to a 150% increase in plant height and tiller number in rice genotypes tested under 0, 50, 100, and 150% of the recommended doses of nitrogen. In general, nitrogen application at any dosage promotes growth in rice, and the optimum amount must be determined and used to prevent excessive nitrogen application that will be destructive to the environment.

*OsNRT1.1b* is a nitrate transporter protein which plays a crucial role in the uptake and transport of nitrate from the soil to the plant. It is a dual-affinity transporter which can operate efficiently under low or high nitrate concentrations, thus allowing rice plants to adapt to varying soil nitrate levels [27,28]. It has been known that *OsNRT1.1b* can distinguish between *indica* and *japonica* rice, and a single base variation in *OsNRT1.1b* explains the difference in NUE between *indica* and *japonica* subspecies [10]. *OsNRT1.1b-indica* exhibits enhanced nitrate uptake compared to *OsNRT1.1b-japonica* rice. An SNP in *OsNRT1.1b* at position 21,759,092 on chromosome 10 showed that *indica* varieties carry the A allele while *japonica* varieties have the G allele. *OsNRT1.1b-indica* showed increased tiller numbers and grain yields [10,27]. This finding is similar to that obtained for the indica varieties tested (PTT1 and HCS), which carry the A allele. In the same study, introducing *OsNRT1.1b-indica* into *japonica* rice improved grain yield by about 10% under normal nitrogen supply and by 30% under 43.75 kg ha^−1^ (N7) nitrogen application. HCS exhibited improved yield per plant with N7 nitrogen application, which was 1.6 times higher than the yield at 87.5 kg ha^−1^ (N14) nitrogen supply. Morphological traits, such as leaf width and high chlorophyll content measured by SPAD, and parameters from hyperspectral images were higher in rice varieties with the G allele, namely, Azucena and LP. Leaf width was found to be largest in tropical *japonica* rice varieties, followed by temperate *japonica*, but the tropical *japonica* was significantly different from the *indica* rice varieties [29,30]. In *japonica* rice, chlorophyll content is an important indicator of growth status [31], where the chlorophyll content is observed based on hyperspectral images of rice canopies. This non-destructive method could be necessary for research for precision agriculture. The indices such as PSRI, SIPI, and MCARI derived from hyperspectral images from the plant phenomics system were, on average, high in the *japonica* varieties. These parameters well differentiate between the *indica* and *japonica* rice varieties and may be good indices of efficient nitrogen use as well.

### 3.2. Trait Relationships for Efficient Nitrogen Use

Nitrogen use efficiency (NUE) was calculated based on biomass yield (BIO), total grain number per panicle (TGN), and yield per plant (YLDPLT). Although PTT1 and HCS, both carrying the A allele at *OsNRT1.1b*, had better NUE than Azucena and LP, which have the G allele of the same gene, HCS, overall, had a better NUE than PTT1. It is proposed that increasing the grain yield of rice from 6.0 to 18.0 t ha^−1^ must depend on increasing biomass and maintaining the harvest index at 0.5 [32]. However, reports are showing that HI is highly variable in crop production, ranging from 0.17 to 0.63 in rice [33,34], and the variation may be due to other factors than increase in biomass. PTT1 showed high biomass accumulation upon nitrogen application at both 43.75 kg ha^−1^ (N7) and 87.5 kg ha^−1^ (N14) due to increased tiller and leaf numbers, while the highest NUE based on biomass yield was found at N7 for HCS and LP. Azucena accumulated biomass at N14. It is interesting to note that the translation to grain yield of assimilates was remarkable in PTT1 and HCS compared with Azucena and LP. Ali et al. [35] found that Azucena has lower NUE compared with IR64 because there is a significant decrease in plant height at low nitrogen levels, which translates into low biomass. Upland rice soils have low nitrogen levels [36], thus explaining the low NUE of Azucena. In addition, between PTT1 and HCS, HCS converts biomass to yield 75% more than PTT1. This indicates poor grain filling in PTT1, which could be attributed to physiology or genetics [37]. HCS had the highest NUE based on total grain number and yield per plant, and, moreover, the ability of HCS to produce high yields was achieved even with (N7) nitrogen fertilization, a manifestation of nitrogen use efficiency.

*OsNRT1.1b* plays a significant role in nitrogen use efficiency in rice because it is involved in the uptake and transport of nutrients within the plant that are essential for plant growth. Since *OsNRT1.1b* functions under both low- and high-nitrogen conditions, the efficiency of nitrogen utilization of HCS could be explained by the function of *OsNRT1.1b* in addition to the efficiency of grain filling, which should be investigated in this rice variety compared with PTT1. Overall, HCS’s possession of the A allele at position 21,759,092 on chromosome 10 contributes to its improved nitrogen use efficiency.

At 43.75 kg ha^−1^ (N7), three principal components explained the total variability in the plants’ performance. PC1 contained most of the agronomic and grain filling traits and parameters related to chlorophyll status. Chlorophyll and pigment parameters were negatively correlated with morphological agronomic traits but positively correlated with grain filling traits, such as paddy seed weight and brown rice weight. Li et al. [38] investigated 7686 rice varieties released in China, and it was identified that traits contributing to grain yield are ecotype-dependent. In the said study, agronomic traits such as leaf, tiller, and grain numbers contributed to grain yield in *indica* varieties, and this was found specifically in HCS, especially at lower nitrogen applications. PCA and the correlation of traits supported this finding, thus contributing to NUE based on yield per plant. The high nitrogen application (87.5 kg ha^−1^ (N14)) resulted in decreased NUE in HCS, which could be due to the decrease in the grain filling rate, by postponing the maximum time of grain filling and prolonging the effective grain filling duration [39]. It could be that the high nitrogen input increased nitrogen concentrations in plant tissue, resulting in a high rate of nitrogen metabolism that enhanced carbohydrate consumption and reduced carbohydrate translocation to grain filling [40]. According to Liang et al. [40], at high nitrogen levels, SPAD values, photosynthesis rates, and stomatal conductance increase and are associated with taller plant heights, more tillers, and larger leaf areas; thus, plants require more carbohydrates to support the plant structures. The correlation of traits and PCA2 in this study also revealed the same associations, and these relationships were found in Azucena at both the 43.75 kg ha^−1^ (N7) and 87.5 kg ha^−1^ (N14) levels. Unfortunately, to support the plant structures, the conversion of absorbed inorganic nitrogen by the roots to organic nitrogen compounds requires carbon skeletons produced by photosynthesis and consequently causes a decrease in carbohydrate accumulation [40] and therefore may decrease yield, as in Azucena. PTT1 has the capacity to increase tiller and leaf numbers and therefore increase biomass yield (see the correlations and the PCA, Figure 10), but these traits were negatively associated with increased leaf width, photosynthetic, and stomatal parameters (PCA, Figure 10); thus, the nitrogen use efficiency of PTT1 is lower than that of HCS. PTT1, a famous rice variety in Thailand, requires more nitrogen to give a better yield, and this is evidence of additional production costs. LP, on the other hand, has a good chlorophyll content and has a good grain filling ability due to having fewer grains per panicle and less assimilate competition, but still the yield per plant was the lowest among the varieties tested and it had the lowest nitrogen use efficiency.

## 4. Materials and Methods

### 4.1. Plant Materials and Cultivation

Rice varieties carrying contrasting alleles of *OsNRT1.1b* at position 21,759,092 on chromosome 10 were identified. The varieties carrying allele A include Pathum Thani 1 (PTT1) and Homcholasit (HCS), while Azucena (Azu) and Leum Pua (LP) carry allele G at *OsNRT1.1b*. These varieties were germinated, and each variety was grown in 12 pots 80 cm in diameter with 3 kg clay paddy field soil (Table S1). The 12 pots were divided into three nitrogen (N) treatments, namely, 87.5 kg ha^−1^ nitrogen (N14), 43.75 kg ha^−1^ nitrogen (N7), and 0 kg nitrogen (N0), with four replications per treatment. The amount of nitrogen applied was based on the recommendations of the Rice Department of Thailand for clay soil and non-photo-period-sensitive rice varieties of 16-20-0 at a rate of 187.5 kg ha^−1^ at 30 days after seeding (DAS) and 46-0-0 at a rate of 125 kg ha^−1^ at 50 to 60 DAS; thus, the normal total nitrogen applied was 87.5 kg ha^−1^. The nitrogen used was NH_4_, while phosphorus and potassium were in the form of P_2_O_5_ and K_2_O, and irrigation was supplied with 5 cm water logging above the soil surface. The plants were grown and weed-controlled by hand until harvest.

### 4.2. Trait Collection

Morphological traits, including total leaf number (TLN), green leaf number (GLN), plant height in cm (PH), tiller number (TN), and leaf width in mm (LW), were collected six times (Timing 1 to Timing 6, or T1 to T6), starting from 30 DAS (seedling) through 45 DAS, 58 DAS, 72 DAS, 86 DAS, and 100 DAS (grain filling). At harvest, panicle length in cm (PL), total grain number (TGN), filled grain number (FGN), biomass yield in g (BIO), yield per plant in g (YLDPLT), weight of 10 paddy seeds in g (PSW), weight of brown rice in g (BRW), and percent seed filling (PGF) were collected.

Physiological traits, including relative chlorophyll content, measured using the Chlorophyll Meter SPAD-502Plus (Konica Minolta, Inc., Tokyo, Japan), and plant F_v_/F_m_ and QY_max (maximum quantum yield of photosystem II), measured using the NSTDA-plant phenomics system (Photon Systems Instruments (PSI), Drasov, Czech Republic), were determined six times (T1 to T6) at 30, 44, 58, 72, 86, and 100 DAS, similar to the morphological traits. Photographs from the system were used to analyze plant phenotypes. The F_v_/F_m_ protocol of the PlantScreen^TM^ Chlorophyll Fluorescence Imaging Unit was used to detect photosynthetic potential with a FluorCam SN-FC800-200 camera (Photon System Instruments, Drasov, Czech Republic). The camera’s distance from the plants was automatically changed. Before capturing fluorescence images, all plants were dark-adapted in a dark tunnel for 30 min. Then, 720 × 560-pixel pictures were taken, yielding four photosynthetic parameter values. The data consisted of minimal fluorescence in the dark-adapted state (F_0_), maximum fluorescence in the dark-adapted state (F_m_), variable fluorescence in the dark-adapted state (F_v_), and maximum PSII quantum yield. The final parameter, QY_max, was employed in this experiment to better understand the basic principles of photosynthesis under the parameters set up [41]. The PlantScreen^TM^ RGB Imaging Unit features a GigE uEye UI-5580SE-C-5 Megapixels QSXGA Camera with a 1/2″ CMOS Sensor (Photon System Instruments, Drasov, Czech Republic) and a 2560 × 1920-pixel resolution. The distance between the camera and the plants was automatically adjusted based on their height. Images were taken in both top and side views. Hyperspectral cameras take images for parameters such as plant senescence reflectance index (PSRI), photochemical reflectance index (PRI), simple ration pigment index (SIPI), and modified chlorophyll absorption in reflectance index (MCARI).

Net photosynthetic rate (*P_n_*), stomatal conductance (*E*), transpiration rate (*g_s_*), and intracellular carbon dioxide concentration (*C_i_*) were measured using a Portable Photosynthesis System (Model LI-6400 XT; LI-COR Biosciences, Lincoln, NE, USA), following the method of Cha-um et al. (2007), and measured at T3 to T6 (58, 72, 86, and 100 DAS). Leaf F_v_/F_m_ and photon yield of PSII (Φ*_PSII_*) were measured using Pulse-Modulated Chlorophyll Fluorescence (Model FMS2; Hansatech Instruments Ltd., Norfolk, UK), according to the protocol of Loggini et al. [42].

Using biomass yield, total grain number, and filled grain number, nitrogen use efficiency (NUE) was determined using the equation below:

NUE = (yield at N14 or N7 − yield at N0)/amount of nitrogen applied (1)

### 4.3. Statistical Analysis

The mean data for each trait were determined for the N0, N7, and N14 treatments using a one-way analysis of variance (ANOVA) analyzed using Jamovi open-access software v.2.6.13 (https://www.jamovi.org; accessed on 27 August 2022). The comparison of means using HSD or Tukey’s Honestly Significant Difference Test was used to find means that were significantly different. PCA was also analyzed using Jamovi. Pearson’s correlation coefficients were used to measure the linear relationship between or among variables to show the strength and direction of the relationship via the R program version 4.3.2 [43].

## 5. Conclusions

An SNP of the gene *OsNRT1.1b*, at position 21,759,092 on chromosome 10, distinguishes between *indica* and *japonica* rice varieties, and the allele in *indica* rice is linked to nitrogen use efficiency (NUE). Homcholasit displayed the highest NUE due to the increased number of grains per panicle and eventually a high yield per plant with 43.75 kg ha^−1^ (N7) nitrogen application for non-photo-period-insensitive rice in Thailand. Unlike Homcholasit, Pathum Thani 1 produced a high biomass yield but was not able to convert assimilates into yield. *Japonica* rice varieties have a smaller number of grains per panicle and showed a high grain filling ability, as exemplified by their having heavier paddy and brown rice seed weights, but due to their having fewer grains per panicle, even under the 87.5 kg ha^−1^ (N14) nitrogen application, the utilization of nitrogen was not efficient. Grain filling in indica rice varieties should be improved to supply sufficient assimilates to the grains without competition due to the high grain number per panicle. The alleles of the *OsNRT1.1b* gene clearly differentiate between plants that are nitrogen-use-efficient and those that are not. Traits such as number of grains per panicle and yield per plant are among the traits that confer nitrogen use efficiency. The markers developed for this gene can be used in selecting breeding lines for nitrogen use efficiency to represent the phenotype associated with NUE.

## Data Availability

The raw data that support the findings of this study are not openly available due to reasons of sensitivity and are available from the corresponding author upon reasonable request. The data are located in a controlled-access database of the Innovative Plant Biotechnology and Precision Agriculture Research Team (APBT) at the National Center for Genetic Engineering and Biotechnology (BIOTEC), Thailand.

## Supplementary Materials

The following supporting information can be downloaded at: https://www.mdpi.com/article/10.3390/plants13202932/s1, Figure S1: Net photosynthetic rates, stomatal conductance, and transpiration rates of four rice varieties (Pathum Thani 1, Homcholasit, Azucena, and Leum Pua) at different growth stages (T3 to T6) and nitrogen supply levels (N0, N7, and N14); Figure S2: Vegetation indices derived from hyperspectral reflectance, plant senescence reflectance index (PSRI), photochemical reflectance index (PRI), structure-insensitive pigment index (SIPI), and modified chlorophyll absorption ratio index (MCARI) of four rice varieties (Pathum Thani 1, Homcholasit, Azucena, and Leum Pua) at different growth stages (T5 and T6) and nitrogen supply levels (N0, N7, and N14); Table S1: Chemical composition of clay paddy soil used in the study.
