# Supplementary material for: Contrasting Alleles of OsNRT1.1b Fostering Potential in Improving Nitrogen Use Efficiency in Rice
Source: Plants (Basel). 2024 Oct 19;13(20):2932. doi: 10.3390/plants13202932 (PMC11510876; doi:10.3390/plants13202932)
Supplement: Supplementary file 1 [file plants-13-02932-s001.zip › plants-3226775-supplementary.pdf]

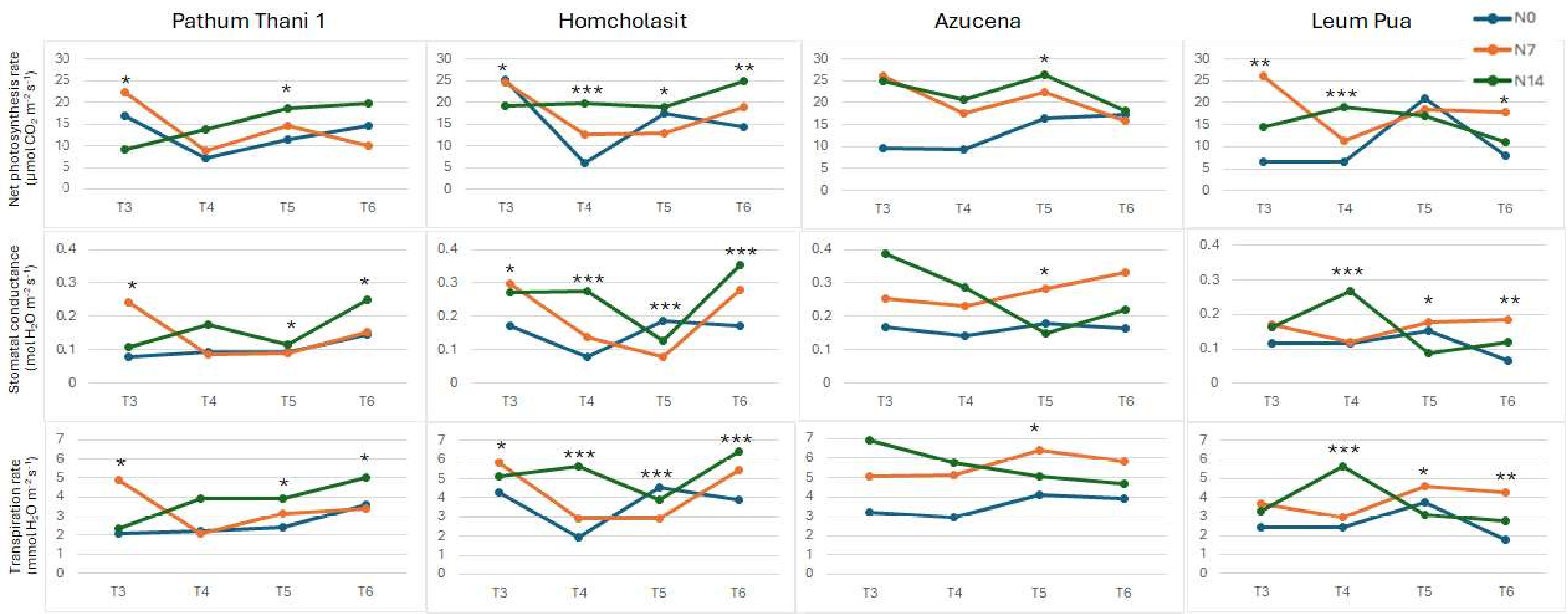

Figure S1. Net photosynthetic rate, stomatal conductance and transpiration rate of four rice varieties (Pathum Thani 1, Homcholasit, Azucena, and Leum Pua) at different growth stages (T3 to T6) and nitrogen supply levels (N0, N7 and N14). *, **, *** represent significant differences at 0.05, 0.01 and 0.001 levels.

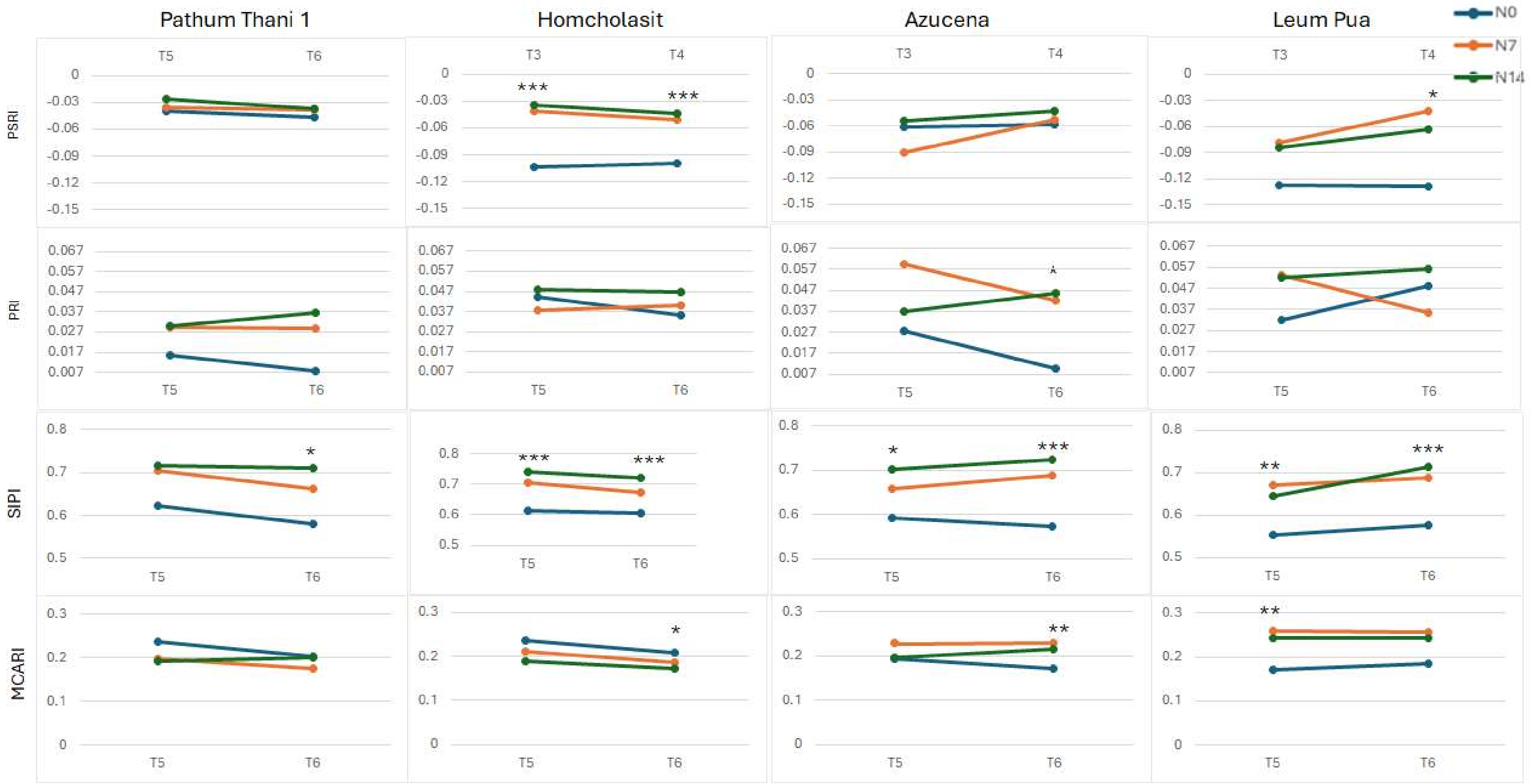

Figure S2. Vegetation indices derived from hyperspectral reflectance, plant senescence reflectance index (PSRI), photochemical reflectance index (PRI), structure-insensitive pigment index (SIPI), and modified chlorophyll absorption ratio index (MCARI) of four rice varieties (Pathum Thani 1, Homcholasit, Azucena, and Leum Pua) at different growth stages (T5 and T6) and nitrogen supply levels (N0, N7 and N14). *, **, *** represent significant differences at 0.05, 0.01 and 0.001 levels.

Table S1. Chemical composition of clay paddy soil used in the study.

Soil analysis	Value
pH (1:1)	6.43
Saturated paste extraction (ECe)	2.11 dS/m
Organic matter (OM)	2.70%
Available P	47.37 mg/kg
Available K	297.73 mg/kg
Available Ca	1486.14 mg/kg
Available Mg	851.58 mg/kg
Total N	13.22 mg/kg
